# Simulation of EO-1 Hyperion Data from ALI Multispectral Data Based on the Spectral Reconstruction Approach

**DOI:** 10.3390/s90403090

**Published:** 2009-04-24

**Authors:** Bo Liu, Lifu Zhang, Xia Zhang, Bing Zhang, Qingxi Tong

**Affiliations:** 1 State Key Laboratory of Remote Sensing Science, Institute of Remote Sensing Applications, Chinese Academy of Sciences, 100101, P.R. China; E-Mails: boxueyu_liu@hotmail.com; zx@irsa.ac.cn; tqxi@263.net; 2 Center for Earth Observation and Digital Earth, Chinese Academy of Sciences, Beijing, 100080, P.R. China; E-Mail: zb@ceode.ac.cn

**Keywords:** Data simulation, hyperspectral data, multispectral data, spectral reconstruction

## Abstract

Data simulation is widely used in remote sensing to produce imagery for a new sensor in the design stage, for scale issues of some special applications, or for testing of novel algorithms. Hyperspectral data could provide more abundant information than traditional multispectral data and thus greatly extend the range of remote sensing applications. Unfortunately, hyperspectral data are much more difficult and expensive to acquire and were not available prior to the development of operational hyperspectral instruments, while large amounts of accumulated multispectral data have been collected around the world over the past several decades. Therefore, it is reasonable to examine means of using these multispectral data to simulate or construct hyperspectral data, especially in situations where hyperspectral data are necessary but hard to acquire. Here, a method based on spectral reconstruction is proposed to simulate hyperspectral data (Hyperion data) from multispectral Advanced Land Imager data (ALI data). This method involves extraction of the inherent information of source data and reassignment to newly simulated data. A total of 106 bands of Hyperion data were simulated from ALI data covering the same area. To evaluate this method, we compare the simulated and original Hyperion data by visual interpretation, statistical comparison, and classification. The results generally showed good performance of this method and indicated that most bands were well simulated, and the information both preserved and presented well. This makes it possible to simulate hyperspectral data from multispectral data for testing the performance of algorithms, extend the use of multispectral data and help the design of a virtual sensor.

## 1. Introduction

Remote sensing is playing an increasingly important role in earth science research and environmental problem solving. A number of earth satellites have been launched to advance our understanding of Earth’s environment. Satellite sensors, both active and passive, capture data from visible to microwave regions of the electromagnetic spectrum. A wide range of satellite data, including multispectral data and hyperspectral data, such as Landsat Thematic Mapper 5/Enhanced Thematic Mapper (TM/ETM+); Global Imager (GLI); Moderate Resolution Imaging Spectroradiometer (MODIS); and Advanced Land Imager (ALI) and Hyperion, are frequently used in oceanography, hydrology, geology, forestry, and meteorology studies. Different studies and applications require different spatial, spectral, radiant resolution, and time-resolution data [1,2]. Hyperspectral sensors monitor hundreds of spectral bands and can provide near-laboratory quality reflectance spectra. The data produced, referred to as hyperspectral data, contain much more information than multispectral data and have greatly extended the range of remote sensing applications [3,4]. Unfortunately, hyperspectral data are much more difficult and expensive to acquire and were not available prior to the development of operational hyperspectral instruments. On the other hand, large amounts of accumulated multispectral data have been collected around the world over the past several decades, therefore it is reasonable to examine means of using these multispectral data to simulate or construct hyperspectral data, especially in situations where the latter are necessary but hard to acquire. Many studies have examined methods to simulate or construct hyperspectral and multispectral data spectra from field spectra or to aggregate spectra of hyperspectral bands into multispectral bands. However, few attempts have been made to simulate hyperspectral data from multispectral data [2, 5–9]. In this paper, we propose a method, based on a spectral reconstruction approach, to simulate hyperspectral data from multispectral data.

Data simulation is widely used in remote sensing. It is often utilized to produce imagery for virtual or new sensors that are in the design stage. Simulated data can be used to assess or evaluate the spectral and spatial characteristics of the sensor, which are critical in the planning of a project [8]. NASA has developed a system to simulate imagery to meet customer needs and costs in a virtual environment (http://www.esad.ssc.nasa.gov/art/). Meanwhile, data simulation is often used to evaluate or assess the influence of different spectral or spatial resolutions on some applications and thus to select the appropriate resolution for a particular problem [10–13]. For example, by simulating hyperspectral data with different spatial resolution, Luo [12] evaluated the adaptability of linear spectral unmixing to different levels of spatial resolution. Jiao [13] simulated hyperspectral data to evaluate the influence of spatial and spectral resolution to vegetation classification. In addition, simulated data are often used to evaluate and test novel algorithms such as target detection and identification algorithms in hyperspectral remote sensing. There is no easy method to simulate hyperspectral data for testing the performance of these algorithms. If simulated hyperspectral data can be easily obtained, it will greatly help the testing and development of new algorithms.

The universal pattern decomposition method (UPDM) is a sensor-independent method which can be considered as a spectral reconstruction approach, in which each satellite pixel is expressed as the linear sum of fixed, standard spectral patterns for water, vegetation, and soil, and the same normalized spectral patterns can be used for different solar-reflected spectral satellite sensors [14]. Sensor independence requires that analysis results for the same sample are the same or nearly the same regardless of the sensor used. Based on this trait, here we present a method based on the universal pattern decomposition method (UPDM) to achieve the goal of simulating hyperspectral data from multispectral data, which can be considered either a method of spectral construction or spectral transform. The hyperspectral and multispectral data are NASA EO-1 satellite/Hyperion and EO-1/ALI data, respectively (see Section 3.2 for a brief introduction). First, we obtained ALI and Hyperion data covering the same area and performed atmospheric correction to obtain surface reflectance data; here Hyperion data served as standard or real data to evaluate the results in the subsequent analysis. Then, we obtained the decomposition coefficients thought to be sensor-independent for the same sample by applying the UPDM to ALI data; these coefficients were subsequently used to construct Hyperion data. Before performing UPDM, standard pattern matrices of both sensors were calculated based on the standard spectral patterns (see Section 2 for details). Finally, the simulated Hyperion data were compared with the real Hyperion data, i.e., test data, to evaluate and assess this method.

## 2. Spectral Reconstruction Approach

### 2.1. Review of the Universal Pattern Decomposition Method (UPDM)

The spectral reconstruction approach is based on the UPDM, which is a sensor-independent method derived from PDM that has been successfully applied in many studies [14–21]. This method can be explained by multi-dimensional analysis, which is also mathematically and practically almost identical to the spectral unmixing method [22–26]. UPDM decomposes reflectance values at each pixel into a linear sum of standard spectral patterns for water, vegetation, soil, and any supplemental patterns using the following formula [20,21]:

(1) $$R_{i}=C_{w}P_{\mathrm{iw}}+C_{v}P_{\mathrm{iv}}+C_{s}P_{\mathrm{is}}+C_{4}P_{i4}$$

Here, *R_i_* is the reflectance of band I measured on the ground or by satellite sensor; *C_w_*, *C_v_*, and *C_s_* are the decomposition coefficients for water, vegetation, and soil, respectively; *C_4_* represents the supplemental coefficients; and *P_w_*, *P_v_*, and *P_s_* are the respective standard spectral patterns for water, vegetation, and soil for some typical sensor captured from the same standard pattern normalized in the same wave region of 350 nm–2500 nm for any sensor, and are therefore related to the properties of each sensor. *P_4_* is the supplementary standard pattern and is an optional component that can be controlled for the purpose of the study.

For each sensor band, the standard spectral patterns of each band *P_i__w_*, *P_i__v_*, and *P_i__s_*, are calculated as follows:

(2) $$P_{\mathrm{ik}}=\frac{\int_{\lambda_{s}\left(i\right)}^{\lambda_{e}\left(i\right)}P_{k}\left(\lambda \right)d\lambda }{\int_{\lambda_{s}\left(i\right)}^{\lambda_{e}\left(i\right)}d\lambda }(k=w,v,s)$$

where *λ_e_*(*i*) and *λ_s_*(*i*) are the start and end wavelengths for band i, respectively, and $\int_{\lambda_{s}\left(i\right)}^{\lambda_{e}\left(i\right)}d\lambda$ is the wavelength width of band i. *P_k_*(*λ*) is the normalized standard pattern, which is fixed for use for all sensors and defined as:

(3) $$P_{k}\left(\lambda \right)=\frac{\int d\lambda }{\int \left|R_{k}\left(\lambda \right)\right|d\lambda }R_{k}\left(\lambda \right)\;\;(k=w,v,s)$$

where *R_k_*(*λ*) represents the spectral reflectance patterns of standard objects and ∫ *dλ* refers to integration of the total wavelength range from 350 nm to 2500 nm. Obviously, *R_k_*(*λ*) satisfies the following normalization equation:

(4) $$\int \left|P_{k}\left(\lambda \right)\right|d\lambda =\int d\lambda \;\;(k=w,v,s).$$

As the supplemental pattern is not fixed, it can be chosen according to the purpose of the study. As an example, we used a yellow-leaf spectrum to briefly show how a supplemental is added. Due to the multi-colinearity, the yellow-leaf pattern cannot be added directly. A residual yellow-leaf pattern is used as the supplementary spectral pattern (see [20]). By analogy to (3), *P_4_*(*λ*) is defined as follows:

(5) $$P_{4}\left(\lambda \right)=\frac{r_{4}\left(\lambda \right)\int d\lambda }{\int \left|r_{4}\left(\lambda \right)\right|d\lambda }$$

where *r_4_*(*λ*) is the residual yellow-leaf value:

(6) $$r_{4}\left(\lambda \right)=R_{4}\left(\lambda \right)-\{C_{w}P_{w}\left(\lambda \right)+C_{v}P_{v}\left(\lambda \right)+C_{s}P_{s}\left(\lambda \right)\}$$

*R_4_*(*λ*) is the measured spectral value for the yellow-leaf sample. For any sensor, *P_i4_* values are calculated using (2) in the same way.

For simplicity, we express UPDM in matrix form as follows [14]:

(7) $$\left(\begin{array}{c} R_{1}\\ R_{2}\\ \vdots \\ R_{n} \end{array}\right)=\left(\begin{array}{cccc} P_{1w} & P_{1v} & P_{1s} & P_{14}\\ P_{2w} & P_{2v} & P_{2s} & P_{24}\\ \vdots & \vdots & \vdots & \vdots \\ P_{\mathrm{nw}} & P_{\mathrm{nv}} & P_{\mathrm{ns}} & P_{n4} \end{array}\right)\cdot \left(\begin{array}{c} C_{w}\\ C_{v}\\ C_{s}\\ C_{4} \end{array}\right)+\left(\begin{array}{c} r_{1}\\ r_{2}\\ \vdots \\ r_{n} \end{array}\right)$$

(8) R=PC+r

where **R** = [*R*_1_, *R*_2_, ⋯, *R*_n_]^T^ is the column vector of observations; n is the number of spectral bands of a sensor; **P** = [*P_w_*, *P_v_*, *P_s_*, *P_4_*]^T^ is the *n* × 4 matrix, called the standard pattern matrix, in which the row vector is the standard spectral pattern for band number n, and P is unique and fixed for a sensor once the standard spectral patterns are given. **C** = [*C_w_*, *C*_v_, *C*_s_, *C*_4_]^T^ is the column vector of UPDM coefficients, and r is the residual column vector. C can be obtained by minimizing the sum-of-squared-error criterion function:

(9) $$\mathrm{Js}={\left\|R-\mathrm{PC}\right\|}_{2}^{2}$$

(10) $$C={\left(P^{\text{T}}P\right)}^{-1}P^{\text{T}}R$$

The reduced χ^2^ employed to evaluate the precision of fitting is defined as follows [21]:

(11) $$\chi^{2}=\sum_{u=1}^{n}r{\left(i\right)}^{2}/\left(n-4\right)$$

### 2.2. A Modification of UPDM

Spectral sensitivity is an important parameter of any sensor and is normally expressed as the spectral response function (SRF), which is the relative responsivity of the sensor to monochromatic radiation of different wavelengths. Various studies have indicated the effects of sensor SRFs on analysis results in a variety of applications, and it is very important to take SRFs into account when comparing data from different sensors and applying physical models [27–31]. To take the effects of SRF into consideration, we revised (2) to calculate standard pattern matrix as follows:

(12) $$P_{\mathrm{ik}}=\frac{\int_{\lambda_{s}\left(i\right)}^{\lambda_{e}\left(i\right)}P_{k}\left(\lambda \right)S_{i}\left(\lambda \right)d\lambda }{\int_{\lambda_{s}\left(i\right)}^{\lambda_{e}\left(i\right)}S_{i}\left(\lambda \right)d\lambda }(k=v,w,s)$$

where *S_i_*(*λ*) represents the SRF of a sensor of band i. The other equations in UPDM remain the same.

## 3. Study Area and Data

### 3.1. Study Area

The study area was located in Yueyang in northeastern Hunan province, P.R. China, where Dongting Lake, China’s second-largest freshwater lake, connects to the Yangtze River. The climate of the Dongting Lake area is between middle and northern subtropical. The annual mean temperature is about 16.4°C–17°C; the mean temperature in January is 3.8°C–4.5°C and in July is about 29°C. The annual precipitation is about 1,100 mm–1,400 mm, with more than half of the rainfall occurring between April and June.

### 3.2. Remote Sensing Data

The remote sensing data used in this study were ALI and Hyperion images. Hyperion and ALI, collecting data over the same area simultaneously, are two of the three sensors onboard the NASA EO-1 satellite with sun-synchronous orbit at an altitude of 705 km. The cross-track width of an ALI scene and a Hyperion scene are 37 and 7.7 km, respectively. The along-track scene length for both ALI and Hyperion will generally be either 42 km or 185 km, depending on the dimensions specified when the scene was scheduled. ALI was built to provide vital information for the next Landsat mission, with 1 panchromatic band and 9 multispectral bands, most of which are comparable to ETM+ bands. The Hyperion sensor collects a total of 242 bands, and its final L1R product provides a total of 198 bands representing 427–2,395 nm continuous spectra with 10-nm spectral resolution.

ALI and Hyperion data covering the study area were acquired on September 2, 2002. The center of the images is 29.38° N, 113.06° E. Frequent torrential rain in May and June 2002 led to localized flooding, which damaged crops and infrastructure along the shores of Dongting Lake. ALI data served as source data from which we attempted to simulate hyperspectral data, whereas Hyperion data served as test data, i.e., real data, to test and evaluate the final results.

## 4. Data Preparation and Spectral Reconstruction

### 4.1. Preprocessing of Remote Sensing Data

Vertical streaks between columns in the along-track direction of image data in a push-broom system are quite common. Such effects are evident in Hyperion data, especially in shortwave infrared (SWIR) channels. To remove the striping, we adopted a statistical balancing method that calculates the mean and standard deviation of a local selectable neighborhood of columns as the reference values to adjust the column data [32]. Local instead of global statistical moments are used to minimize changes made before atmospheric correction as global statistical destriping may alter mid-frequency spatial effects [32, 33]. Only a small subset of bands with the most severe streak effects was selected for such destriping. The intention here was still to minimize changes, although this conservative treatment may not remove striping effects fully.

After destriping, Hyperion L1R data were atmospherically corrected using ACORN5.0 in mode 1.5 to obtain surface reflectance data. As ALI data are of very good quality, atmospheric correction was performed directly using ACORN5.0 in mode 5.0 without similar preprocessing.

Although both sensors are onboard the same satellite, the areas covered by the pixels of their images were not strictly identical. Hyperion data were geometrically corrected to ALI data using first-order polynomial interpolation and bilinear resampling.

### 4.2. Obtaining Standard Pattern Matrix

As described in Section 2, it is necessary to calculate standard pattern matrices of ALI and Hyperion to apply UPDM. According to (12), SRFs of both sensors are also needed. The SRFs of ALI are available from the website of CSRIO (http://www.eoc.csiro.au/hswww/oz_pi/specresp.htm). We use the Gaussian function to simulate the SRFs for Hyperion. The Gaussian function is widely accepted for many detector instruments and is reasonably constant across detector arrays within the same optical system [34].

The Gaussian function g(λ̄_i_, σ_i_) can be represented by the central wavelength λ̄_i_ and the bandwidth σ_i_, which is a function of Full Width at Half Maximum (FWHM) (14). With the assumption that the peak of the Gaussian function corresponding to the central wavelength is 1, the formula of g(λ̄_i_, σ_i_) is given as:

(13) $$g\left({\bar{\lambda }}_{i},\sigma_{i}\right)={\text{exp}}^{-\frac{{\left({\bar{\lambda }}_{i}-\lambda \right)}^{2}}{{2\sigma }^{2}}}$$

(14) $$\sigma_{i}=\frac{{\mathrm{FWHM}}_{i}}{2\sqrt{2\;\text{ln}\;2}}$$

where the subscript i represents the band i of a sensor. All SRFs of Hyperion can be well constructed in this way involving two variables, i.e., central wavelength and FWHM, which are known parameters.

After obtaining the SRFs of both sensors, the standard pattern matrices are calculated using (12). The standard spectral patterns used here were the same as those used previously [20]. The standard pattern matrix of ALI, denoted as **P**_A_, has an order of 9 × 4, and the matrix of Hyperion, denoting as **P**_H_, has an order of 106 × 4. That is, information from all ALI multispectral bands is used to simulate Hyperion data. Due to the strong water vapor absorption, low SNR, and valid region of wavelength of standard spectral patterns used, a subset of 106 Hyperion bands were used in this study (Table 1).

### 4.3. Simulating Hyperion Data Based on UPDM from ALI Data

UPDM is applied to ALI data to acquire the decomposition coefficients vector **C**_A_, which is considered to be sensor-independent, i.e., it holds the same value when UPDM is applied to Hyperion data:

(15) $$R_{A}=P_{A}C_{A}+r$$

(16) $$C_{A}={\left(P_{A}^{T}P_{A}\right)}^{-1}P_{A}^{T}R_{A}$$

where subscript A denotes ALI.

To construct Hyperion data, we substitute **C**_A_ for **C**_H_ in the following equation:

(17) $$\begin{array}{ll} R_{H} & =P_{H}C_{H}\\ & =P_{H}C_{H}\\ & =P_{H}{\left(P_{A}^{T}P_{A}\right)}^{-1}P_{A}^{T}R_{A} \end{array}$$

where subscript H similarly denotes Hyperion.

## 5. Results and Discussion

Following the process flow discussed above, 106 bands were simulated based on UPDM from ALI data. We used source data referring to ALI data, simulated data referring to the new generated Hyperion data, and original data or real data referred to as real Hyperion data. Here, we evaluated this simulating method by comparing simulated and original data with regard to three aspects. First, the general appearances of both types of data were compared by visual interpretation to determine whether they have similar visual effects. Second, the statistical characteristics of both types of data were compared to determine whether they show a good correlation. Finally, we performed classification of both types of data to evaluate how information is preserved in application.

### 5.1. Comparing Simulated Hyperion Data and Real Hyperion Data by Visual Interpretation

**Figure 1.**
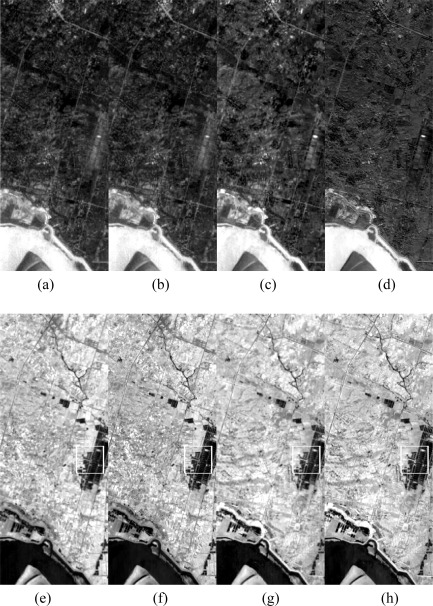
Visual comparison of simulated Hyperion data and original Hyperion data: (a) and (b) are band 13 of original Hyperion data and simulation Hyperion data, respectively; (c) and (d) are band 19; (e) and (f) are band 94; (g) and (h) are band 148. There are no significant difference between simulated and original data of band 13, 94 and 148 by visual interpretation while band 19 shows some obvious differences.

Based on UPDM, we used all nine bands of ALI data to simulate the final set of 106 bands of Hyperion data. According to the correlation coefficients between each pair of original and simulated bands, we selected four bands, band 13 (average central wavelength 477.69 nm), band 19 (538.74 nm), band 94 (1,083.99 nm), and band 148 (1,628.81 nm), to show their general appearance by visual interpretation (See Section 4.2 and Figure 4 for detailed discussion of correlation). Specifically, the wavelength of band 94 is not covered by any ALI band. Therefore, we wish to show not only those Hyperion bands covered by ALI bands that can be explained as divided from the ALI band, but also those that are uncovered, which are simulated or “created.” Band 13, band 94, and band 148 had higher correlation coefficients (0.98, 0.97, and 0.96), which indicate well-simulated bands, whereas band 19 had a lower correlation coefficient of 0.88, indicating a minor fraction of bands that are not simulated as well as others (Figure 1).

**Figure 2.**
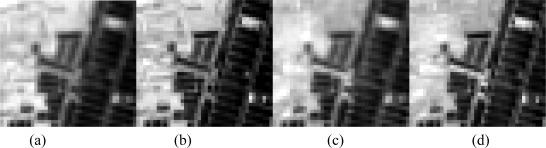
Visual comparison of the magnification of a detailed region circled by the white rectangle in Figure 1. (e)–(h): (a) and (b) are band 94 of original Hyperion data and simulated Hyperion data; (c) and (d) are band 148. The texture of the pond, the pattern of vegetation distribution on the left part of this region, several little bright objects on the lower right part, the border between pond and land keep consistent and look same between original data and simulated data. Original data is a bit vaguer because of the resampling method in geometrical correction.

By interpreting each pair of images in Figure 2, we can see no significant differences between the simulated and original data for band 13, band 94, or band 148. The tone, pattern, texture, shape, and border of objects in both images all look quite similar with no obvious differences. These observations indicated that, from the viewpoint of visual interpreting, the information of ground objects and features presented in real data were also well preserved and presented in our simulated Hyperion data. The high degree of similarity and coherence of band 94 showed that our method could well simulate even those bands uncovered by multispectral data. Band 19 showed lower degrees of similarity and identity, with the most obvious differences in the color of objects; however, the texture and shape were still consistent and coherent. The most obvious differences were related to some parts of the vegetation area. These differences may have been because our standard spectral pattern of vegetation was not collected in the study area and may therefore not accurately present the characteristics of vegetation in this area around the wavelength of band 19. It is thought that the simulated results would have been much better if field spectra for this area during the same period had been available.

For comparison of detailed regions, we selected a small area covering the border of the pond circled by the rectangle in bands 94 and 148. This small region had a great deal of variety and many details and therefore served as a good test region (Figure 2). Similar to the observations discussed above, most details and variety were also well presented in the simulated data. For example, the texture of the pond, the pattern of vegetation distribution on the left part of this region, several small bright objects in the lower right part, and the border between the pond and land remained consistent and coherent between the original and simulated data. The original data often appear slightly vaguer than the corresponding simulated data. This is attributable to the geometrical correction in which the bilinear resampling method can degrade the spatial resolution of the original Hyperion data. However, this effect cannot be removed completely, as instantaneous fields of view of both sensors are not completely identical.

### 5.2. Comparing Simulated and Real Hyperion Data by Statistical Analysis

The reflectance of original 9-band ALI multispectral data and the simulated 106-band Hyperion data from ALI is obviously different. However, the reflectance of original Hyperion data and that of the simulated Hyperion data is very similar, the latter spectral curve are a little more smooth than that of the former.

**Figure 3.**
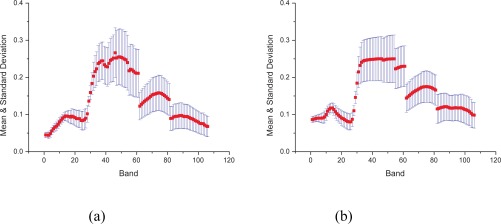
Mean and standard deviation of 106 bands of original and simulated data: (a) shows the mean and standard deviation of original bands, and (b) shows the mean and standard deviation of simulated bands (for convenience in plotting, we used the sequence numbers 1–106 to refer to bands. The corresponding bands are listed in Table 1).

For simple statistical comparison, we used the mean and standard deviation of each band from both datasets (Figure 3). Although the mean and standard deviation of each pair of bands of both datasets showed a large degree of variation, we still obtained some good observations. For most bands, the trends of variation in the mean and standard deviation and their dynamic ranges were similar.

**Figure 4.**
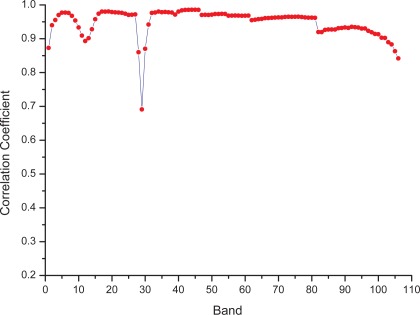
Correlation coefficients between simulated and original Hyperion data of 106 bands.

We also calculated the correlation coefficients between each band of original data and the counterpart in the simulated data (Figure 4). As shown in Figure 4, most bands had correlation coefficients >0.95 (n = 70, bands 10–16, 22–34, 39–53, 87–94, 107–113, and 139–158), and a small fraction had correlation coefficients <0.90 (n = 9, bands 8, 19, 35–37, and 216–219); among the latter, band 36 had a the lowest correlation coefficient of 0.69. A higher coefficient suggested much better simulation of the corresponding band, whereas a coefficient lower than 0.90 indicated poor simulation.

**Figure 5.**
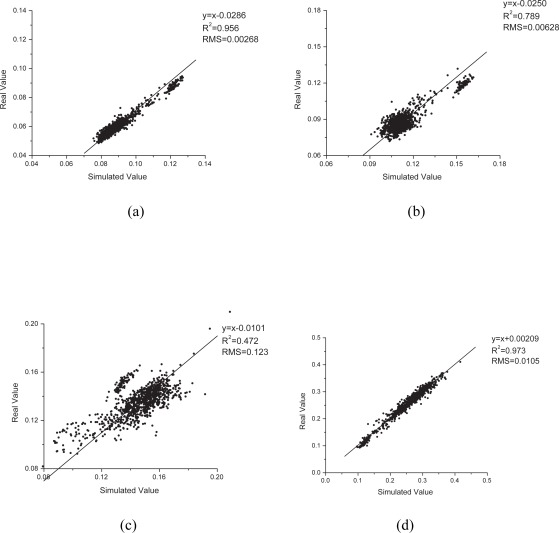

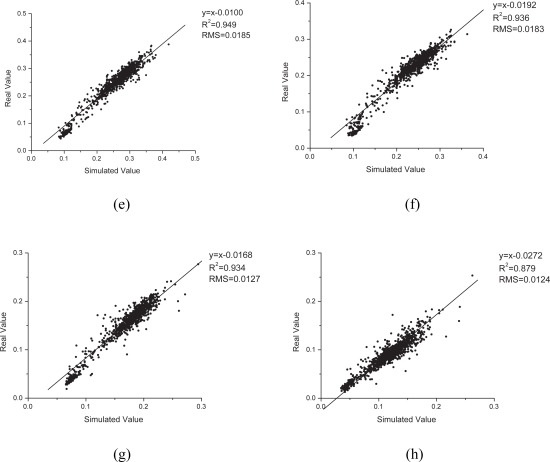
Linear regression analysis of simulated and original data. Charts from (a) to (h) are for bands 13, 19, 36, 52, 94, 113, 148, and 208, respectively.

To further evaluate this method, we sampled 1000 pixels at random to perform linear regression with a fixed slope of 1 on a subset of bands, which corresponded to different levels of correlation (Figure 5). These were band 13 (correlation coefficient 0.98, average central wavelength 477.69 nm), band 19 (0.88, 538.74 nm), band 36 (0.69, 711.72 nm), band 52 (0.99, 864.35 nm), band 94 (0.97, 1,083.99 nm), band 113 (0.96, 1,275.66 nm), band 148 (0.96, 1,628.81 nm), and band 208 (0.92, 2,234.12 nm). Generally, the linear fitness of these bands was consistent with the band correlation coefficient. They could be assigned into three groups according to the fitness performance. The first group included bands 19 and 36, with correlation coefficients <0.90. Their data points showed loose scattering and did not cluster tightly around the fitted line, and their R^2^ values (0.789 and 0.472, respectively) were much lower than those of other bands. As indicated by its lowest correlation coefficient, band 36 showed the poorest performance with the greatest RMS of 0.123 and lowest R^2^ of 0.472. The wavelength of band 36 was 711 nm, corresponding to the red edge. The rapid change in vegetation reflectance around the red edge may degrade our model performance and cause the lowest correlation coefficient and poor performance. This may also have been responsible for the low correlation coefficients of bands 35 and 37, as they were also around the red edge. Adding some supplementary spectral patterns accounting for this rapid change into UPDM or replacing the vegetation standard spectral pattern with ground-measured vegetation spectra in the study area may improve the results. The second group consisted of bands 52, 94, 113, 148, and 208, all of which had correlation coefficients >0.9. The data points for these bands clustered around the fitted line very well with quite high R^2^ values (0.973, 0.949, 0.936, 0.934, and 0.879, respectively), and their fitted lines were very close to the line 1:1. These observations indicated that these bands were well simulated and highly similar to the original bands. The best performance was observed for band 52; its fitted line was y = x + 0.00209 (R^2^ = 0.973), indicating that the simulated band was almost the same as the real data. Band 13 alone was considered a separate group for which the intercept of the fitted line (0.0286) relative to the dynamic range of data (0.06–0.13) was much greater than that for the second group, causing it to move away from the line y = x. However, the data points of band 13 fit the line quite well. The high values of R^2^ (0.956) and correlation coefficient (0.98) also suggested good linearity.

**Figure 6.**
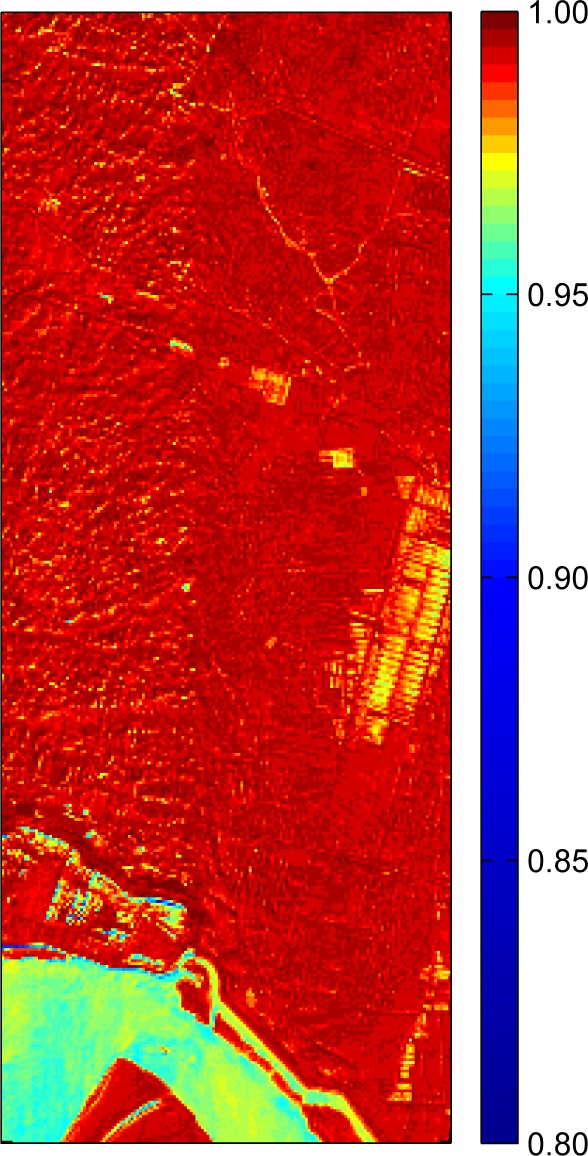
Cosine values of vector angles between simulated and original data of each pixel.

To evaluate the general results of the whole dataset, the vector angles between simulated and original data of each pixel were calculated. The images were displayed as cosine values of the angle, and a higher value corresponded to a smaller angle (Figure 6). Most pixels reached values >0.95, especially for the deep red area. We also noted that the areas in yellow and canyons had lower values, indicating slightly greater differences between simulated and original data. Interestingly, these areas all corresponded to river or pond areas. Water has much lower reflectance than land, especially in the infrared region. This results in a lower SNR of data in water areas, and the noise may lead to poorer simulation. This result was similar to the finding in [16]. Some borders of different ground objects or line features with narrow width also have lower values caused by the resampling method.

Combining the above discussion and analysis, most simulated bands, with the exception of a small fraction with quite low correlation coefficients, showed strong correlations and high linearity with the original bands. The vector angle image also showed a high degree of similarity and good simulation for most areas. These observations indicated that our method is valid for simulating Hyperion bands from the viewpoint of statistics.

### 5.3. Comparing Simulated Hyperion Data and Real Hyperion Data by Classification Application

**Figure 7.**
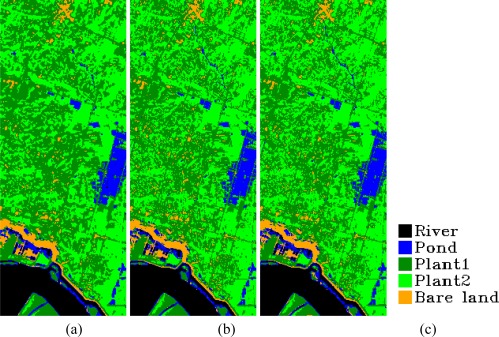
Classification results of original Hyperion data, simulated Hyperion data, and ALI data: (a) shows the classification image of original Hyperion data, (b) shows the classification image of simulated Hyperion data, and (c) shows the classification image of ALI data. (Class label “pond” refers to water, which has characteristics of pond water; label “plant 1” refers to sparse plant area; and Label “plant 2” refers to dense plant area).

We also performed classification using the spectral angle mapper method on the whole original Hyperion data (106 bands), simulated Hyperion data (106 bands), and ALI data (9 bands) to evaluate the general performance of our simulation method in classification application (Figure 7). That is, we wish to determine whether, being reconciled with the hyperspectral data, simulated data can preserve the information of ALI data.

The aim of our method is to simulate hyperspectral data from multispectral data to make it comparable to the real hyperspectral data. Our method involves transformation to present the information of multispectral data in the new simulated hyperspectral data, but not creating or adding new information. This is quite reasonable, as we could never create any new information just using mathematical techniques. The situation here is quite similar to the PCA method, which never creates any new information but reassigns inherent information.

From the classification images, we can see that the classification results on simulated data are similar to those on ALI data, indicating that the information of ALI data is well preserved in the simulated data using our method. That is, the inherent information of ALI data is not lost after being reassigned. In addition, the classification on original Hyperion data is also similar to the results of simulated Hyperion data shown by the overall classification accuracies, which are calculated using the classification results on original Hyperion data as the reference image to compare and evaluate the classification results of simulated Hyperion data and ALI data (Table 2). The overall accuracy of the classification on simulated data was 87.6%, and the kappa coefficient was 0.808, whereas the accuracy on ALI data was 86.8% and its corresponding kappa coefficient was 0.797.

The classification results showed that our method successfully preserved the inherent information and presented it in the new data.

## 6. Summary and Conclusions

We have proposed a method to simulate hyperspectral data from multispectral data based on the spectral reconstruction method UPDM. A total of 106 bands of Hyperion were simulated from ALI data covering the same area. Visual comparison showed that the simulated data successfully presented the information of ground features and objects described by the original data for interpretation. To further evaluate our method, we compared the simulated and original data by statistical methods. The results indicated that most bands had very high correlation coefficients, suggesting a high degree of similarity and good consistency of the simulated bands to the original bands. The detailed results of linear regression analyses further verify that, for the bands with high correlation coefficients, the data points were generally clustered very tightly and fit the line 1:1 very well. These observations indicated that most bands showed good linearity and similarity to the original data. The high cosine values of the vector angle between the simulated and original data of each pixel also demonstrated the general good performance of our method.

However, a small fraction of bands showed lower correlation coefficients, corresponding to poor simulation. This may have been because our standard spectral patterns were not collected in the study area. It may be possible to improve the results by adding supplementary patterns and replacing the standard spectral patterns with those derived from ground-measured spectra.

The aim of our method is to simulate hyperspectral data from multispectral data and make them comparable to the real hyperspectral data. Similar to PCA, our method attempts to preserve and reassign the inherent information of multispectral data to the simulated data and to make full use of them, but not to add or create extra new information. The similarity between classification results derived from ALI data and simulated Hyperion data showed that the inherent information of ALI data were well preserved by reassignment to simulated Hyperion data.

Simulated data could serve as a powerful tool in algorithm testing and assessment and could act as a potential surrogate when real hyperspectral data are unavailable. Validation and evaluation of such algorithms should be conducted using hyperspectral images covering a wide range of spatial complexities, but acquiring enough hyperspectral data to meet this need can be difficult. Our method can provide simulated hyperspectral imagery with the spatial complexity of real-world imagery, thus allowing for extensive yet lower-cost testing of algorithms over a wide variety of environmental conditions. In addition to algorithm development and testing, our method can also be applied to simulate the imagery of new sensors still in the design stage.

Although this pilot study demonstrated the good general performance of our method from the viewpoint of visual interpretation, statistical comparison, and classification application, further studies of both the theory and applications should be performed to improve this method. For example, we may add some supplementary spectral patterns or consider the variability of standard spectral patterns, attempt to derive standard spectral patterns from ground-measured spectra, and select different standard spectral patterns for different applications.

## Figures and Tables

**Table 1.** Hyperion 106-band subset used in this study.

**Sequence NO.**	**Bands**	**Wavelengths (nm)**
1–46	8–53	427–885
47–54	87–94	1,013–1,084
55–61	107–113	1,215–1,276
62–81	139–158	1,538–1,730
82–106	195–219	2,103–2,345

**Table 2.** Classification accuracies of simulated Hyperion data and ALI data using the classification results of original data as reference image.

	**Simulated Hyperion**		**ALI**	
	
**Class**	**Product Accuracy (pixels)**	**User Accuracy (pixels)**	**Product Accuracy (pixels)**	**User Accuracy (pixels)**
River	4,957/5,092	4,957/5,156	4,965/5,092	4,965/5,195
Pond	2,838/3,473	2,838/3,479	2,883/3,473	2,883/3,584
Plant1	24,713/28,839	24,713/27,835	23,896/28,839	23,896/26,669
Plant2	21,448/23,997	21,448/24,366	21,769/23,997	21,769/25,681
Bare Land	2,076/2,599	2,076/3,164	2,042/2,599	2,042/2,871
Kappa	0.808		0.797	
Overall	87.6%		86.8%	
